# Trajectories of Sensory Over-Responsivity from Early to Middle Childhood: Birth and Temperament Risk Factors

**DOI:** 10.1371/journal.pone.0129968

**Published:** 2015-06-24

**Authors:** Carol Van Hulle, Kathryn Lemery-Chalfant, H. Hill Goldsmith

**Affiliations:** 1 Waisman Center, University of Wisconsin-Madison, Madison, Wisconsin, United States of America; 2 Department of Psychology, Arizona State University, Tempe, Arizona, United States of America; 3 Department of Psychology, University of Wisconsin-Madison, Madison, Wisconsin, United States of America; University of Portsmouth, UNITED KINGDOM

## Abstract

Sensory over-responsivity, a subtype of sensory modulation disorder, is characterized by extreme negative reactions to normative sensory experiences. These over-reactions can interfere with daily activities and cause stress to children and their families. The etiology and developmental course of sensory over-responsivity is still largely unknown. We measured tactile and auditory over-responsivity in a population-based, typically developing sample of twins (N=978) at age two years via a caregiver report temperament questionnaire and again at age seven years via a sensory over-responsivity symptom inventory. Participating twins were treated as singletons although all analyses controlled for clustering within families. Children were divided into four trajectory groups based on risk status at both ages: low symptom (N=768), remitted (N=75), late-onset (N=112), and chronic (N=24). A subset of children who screened positive for SOR in toddlerhood (N = 102) took part in a pilot study focused on sensory over-responsivity at four years of age. Children in the chronic group had more severe symptoms of sensory sensitivity at age four years, including more motion sensitivity, than the other trajectory groups. Children in the chronic group had a younger gestational age and were more likely to be low birth-weight than the low symptom group. Differences between remitted and late-onset groups and the low-symptoms group were inconsistent across measures. Sensory over-responsivity was modestly correlated across ages (*r* = .22 for tactile over-responsivity and *r* = .11 for auditory over-responsivity), but symptoms were more stable among children born prematurely or who had more fearful and less soothable temperaments. A clear implication is that assessment over development may be necessary for a valid sensory processing disorder diagnosis, and a speculative implication is that sensory over-responsivity symptoms may be etiologically heterogeneous, with different causes of transient and stable symptoms.

## Introduction

Individuals vary widely in their reactions to sensory stimuli, with some children and adults reporting aversive, even painful, responses to contact with everyday objects and exposure to everyday sounds. Some people find the sound of vacuum cleaners or sirens highly aversive, and some children fuss about stiff new clothes and labels sewn inside collars. They may dislike being lightly touched or vigorously protest brushing their teeth. Extremes of these behaviors can prove stressful, especially for children and their families [1]. These types of “over-responsive” reactions are consistent with an underlying Sensory Processing Disorder. Sensory Processing Disorder may also increase risk for developing other childhood behavior problems [2,3], yet our understanding of the development of these disorders across the lifespan is limited. Our goal was to examine the development of symptoms of sensory over-responsivity in a community-based sample of typically developing children.

## Definition and Background

Ayres [4] first identified patterns of perceptual-motor dysfunction in young children with learning disabilities in the 1960s and coined the term, tactile defensiveness. As the nosology evolved, different terms were used to describe various types of sensory impairment, including sensory defensiveness, sensory integration disorder, and sensory modulation disorder. Miller et al. [5] proposed the umbrella term Sensory Processing Disorder (SPD) to encompass the three subtypes of sensory impairment: sensory modulation disorder either over-responsivity to typical sensations or under-responsivity and sensation seeking (SMD), sensory-based motor disorder (SBD), and sensory discrimination disorder (SDD). The most common form of SMD is Sensory Over-Responsivity [6], usually to auditory and/or tactile sensations [7]. Miller et al. [5] defined sensory over-responsivity (SOR) as responses that were rapid in onset, prolonged, and greater in intensity compared with peers. Many studies pool results across subtypes. We use the term SOR where results are specific to the over-responsivity subtype, and the term SMD if the results are pooled or if it is unclear which subtype was targeted.

SMD likely reflects differences in neurological structures that support the processing and integration of sensory information. Dunn and colleagues [8] provided a framework that connects the behaviors associated with sensory processing dysfunction to neurological dysfunction in habituation and sensitization. Individuals with a high neurological threshold are expected to react less readily to stimuli and may seek more intensive stimuli, whereas individuals with a low threshold may react too readily and become easily overwhelmed and thus withdraw from stimulation. In this framework, the SOR subtype of SMD represents an inability to balance the detection of novel stimuli against habituation to on-going stimuli. That is, individuals with SOR may be unable to adequately attenuate irrelevant stimuli (e.g. hum of fluorescent lights) once the stimulus is detected [9].

Exaggerated responses to typical sensation can interfere with daily activities [10] and contribute to family stress [11]. Thus, SOR has long been identified as an important area of research and treatment in pediatric occupational therapy. Sensory sensitivity (similar to SOR) was included in the Diagnostic Classification:0–3 [12] and the classification of the International Council of Developmental and Learning Disorders [13]. However, debate continues as to whether sensory disorders are truly distinct diagnostic entities [14]. SPD (and all subtypes) was ultimately excluded from the most recent edition of the DSM [15] due to questions over the validity of the disorder.

Much of the research on SPD has focused on children with developmental disabilities or other childhood disorders. Children with developmental disabilities display higher rates of sensory dysfunction than typically developing children, especially children with autism [16,17], Fragile X [18], and attention deficit hyperactivity disorder [19,20].

To examine the utility of treating SMD as a distinct from other disorders where sensory dysfunction is a common occurrence, we focus on sensory experiences among the typically developing population. Evidence suggests a continuum of SMD symptoms in both typically developing children and adults, with some scoring in the extreme range [10,21,22]. Goldsmith, Van Hulle, Arneson, Schrieber, and Gernsbacher [23], reported that Sensory Over-responsivity symptoms were associated with fearful temperament and anxiety in toddlerhood but were largely distinct from other common childhood disorders. Furthermore, differences exist between children with SMD in the absence of other disabilities and typically developing children in both neurological function [9,24] and structure [25]. For instance, McIntosh et al. [6] reported that children with SMD had larger initial electrodermal responses and slower habituation to repeated sensory stimuli. In a recent study, children diagnosed with SPD exhibited less white matter integrity in areas associated with sensory detection and integration compared with matched, neurotypical controls [25].

### Prevalence rates

Several obstacles impede obtaining accurate prevalence rates outside of clinical populations. No “gold standard” assessment for sensory processing disorders exists [26], and most studies rely on questionnaires rather than diagnosis by a trained clinician.

A variety of parent, teacher- and self-report questionnaires target different age groups and populations (clinical vs.non-clinical), tap different aspects of sensory dysfunction, and are used in different settings (clinical practice vs re.search). The Sensory Over-responsivity Inventory [27] which focuses on over-responsivity to typical sensations, and the suite of Sensory Profile questionnaires [28], which assess four dimensions of sensory processing based on neurological threshold (high or low) and self-regulation strategy (passive or active) in multiple sensory domains across the lifespan, are two instruments often used in studies of typically developing children. Sensory questionnaires rarely have established criteria for identifying children at-risk for SOR. Only the Sensory Profile questionnaires have published norms. Given heterogeneity in assessments and criteria, the similarity of prevalence rates for the SOR subtype across studies of similarly aged participants is striking.

An estimated 5–13% of 703 kindergarteners met criteria for SOR via the Sensory Profile, depending on whether non-responders were assumed to be unaffected or not [29]. Children met criteria if they scored 3–4 standard deviations below the mean based on published norms. Another study of school-aged children examined the prevalence of *elevated* sensory symptoms (i.e., at-risk for SOR), based on presence of four or more tactile or auditory symptoms on the Sensory Over-responsivity Inventory [10]. An estimated 16.5% of 925 participants fell in the elevated range. Using the same measure, Van Hulle et al. [22] reported that 20.7% of school-aged twins similarly exhibited elevated sensory over-responsivity symptoms. A recent study of adult self-reports of the Sensory Profile found 24.3% fell into the “higher than most people” range (based on published norms) on the dimension of sensory sensitivity [30]. Another study reported that 22.5% of 19–64 year olds and 26.6% of those 65+ years old fell into the “higher than most people range” for the dimension of sensory sensitivity [31].

Similarity in prevalence rates between the studies reviewed above is almost certainly due at least in part to the similarity in the study participants (largely Caucasian and middle class). In contrast, Reynolds, Shephard and Lane [32] found that 35% of participating minority children enrolled in a Head Start program could be classified as having an SMD using the Short Sensory Profile, translating to an overall prevalence rate of 17% under the conservative assumption that all non-participants did not have an SMD. One fourth of participating children scored in the “definite difference” range on tactile sensitivity.

### Development

Compared with the prevalence rates for common mental health disorders, which generally range from 2–15% (lifetime diagnosis) for children/adolescents and 2–9% (12 month diagnosis) for adults [33], the prevalence rates cited above appear unrealistically high. Thus, individuals who score in the at-risk or “higher than most people” range likely represent a heterogeneous mix of individuals, some with an underlying sensory processing disorder and some without. Individuals who experience many sensory symptoms throughout their lives are more likely to have a true underlying disorder. Yet most of the research identifying and characterizing otherwise typically developing children with SPD has been conducted without reference to developmental changes. Do symptoms persist over time or are they largely transient? How might neurological differences interact with other non-biological factors to influence development of SOR symptoms?

A small group of regulatory-disordered and typically-developing infants was followed from 8–11 months to four years of age [34]. One out of 13 typically-developing infants exhibited vestibulatory-based sensory integration deficits at age four years while eight out of nine regulatory-disordered infants continued to exhibit a range of problems, including poor motor planning, SOR, and inattention/hyperactivity. Ben-Sasson, Carter and Briggs-Gowan [35] measured SOR symptoms via parent report for 521 typically developing children at ages one, two and three years and again at age seven years. They examined continuity in symptoms over the entire spectrum and within the clinical range. Children who started with more severe SOR symptoms and/or experienced an increase in symptoms from age one to three years had more severe symptoms at age seven years. Moreover, 33.3% to 53.8% who fell above clinical cut-off at ages one, two or three years had elevated SOR symptoms at age seven, which suggests that a subset of children experience chronic symptoms. More research is needed to establish the developmental course of SOR symptoms, not just among individual who experience elevated symptoms but across the range of symptom severity.

### Pregnancy, birth, and temperament correlates

Identifying factors that differentiate children with transitory versus chronically-elevated symptoms or that contribute to stability across the symptom range is crucial to understanding the processes that underlie sensory over-responsivity symptoms. Pregnancy and birth related experiences are implicated as possible risk factors for sensory processing disorders. Prenatal exposure to certain medications, alcohol, or stress are associated with an increase in symptoms of sensory processing disorders in both animals [36,37] and humans [38]. May-Benson, Koomar and Teasdale [39] reviewed birth records for 1000 children ages 3–17 years diagnosed with some type of sensory processing disorder. Approximately 40% of mothers experienced some health-related issue or stress during pregnancy, with the incidence of several birth complications such as prolonged labor, fetal distress and jaundice higher than the national average. Shorter gestational age and prematurity are associated with more frequent behaviors indicative of the SOR subtype in infants and toddlers [23,40]. In an earlier report with our twin sample, prematurity, low birth-weight, and obstetrical complications were associated with higher SOR symptoms in toddlerhood [38].

Throughout the lifespan, individuals with many sensory over-responsivity symptoms can be differentiated from more typical peers on temperament dimensions [23,41]. Rothbart’s popular conceptualization of temperament incorporates sensory processing symptoms in two factorial domains, effortful control (sensory regulation) and negative affect (sensory reactivity). However, a recent study suggests that sensory symptoms compose a correlated but distinct third domain [42]. Other studies confirm that symptoms of the SOR subtype tend to be negatively correlated with regulatory aspects of temperament such as inhibitory control and soothability, and positively correlated with negative affect such as anger, sadness, and fear [23,40,43].

No longitudinal studies of SOR incorporate measures of prenatal/birth complications or temperament as predictors of SOR symptoms over time. Therefore, we do not know whether these associations persist over time, or can be used to distinguish different symptom trajectories. Twins are more likely than singletons to be born prematurely or to experience birth complications [44,45], yet twins are largely indistinguishable from their singleton peers on measures of personality and behavior problems [46,47]. Thus, twins provide an enriched sample for examining the effects of birth complications without confounding any potential relationship between temperament and SOR.

Our study has three aims. First, as noted above, prevalence rates based on a single assessment may include individuals with transient symptoms. Therefore, we characterized trajectories of risk status for the SOR subtype in a sample of twins from age two years to age seven years to determine the rate of *chronic* elevated SOR symptoms. We anticipated that, while a core group of children would maintain a high number of symptoms over time, sensory symptoms would fluctuate for most children. Second, we examined potential predictors of experiencing chronically elevated symptoms. That is, do children with chronically elevated symptoms have different etiology/risk factors than children with transient symptoms? We hypothesized that temperament dimensions related to regulation and negative affect and prenatal/birth complications would distinguish trajectory groups. Third, we examined temperament and prenatal/birth complications as potential moderators of the stability of SOR symptoms from two to seven years across the spectrum of symptom levels.

## Methods

### Participants

**Fig 1:**
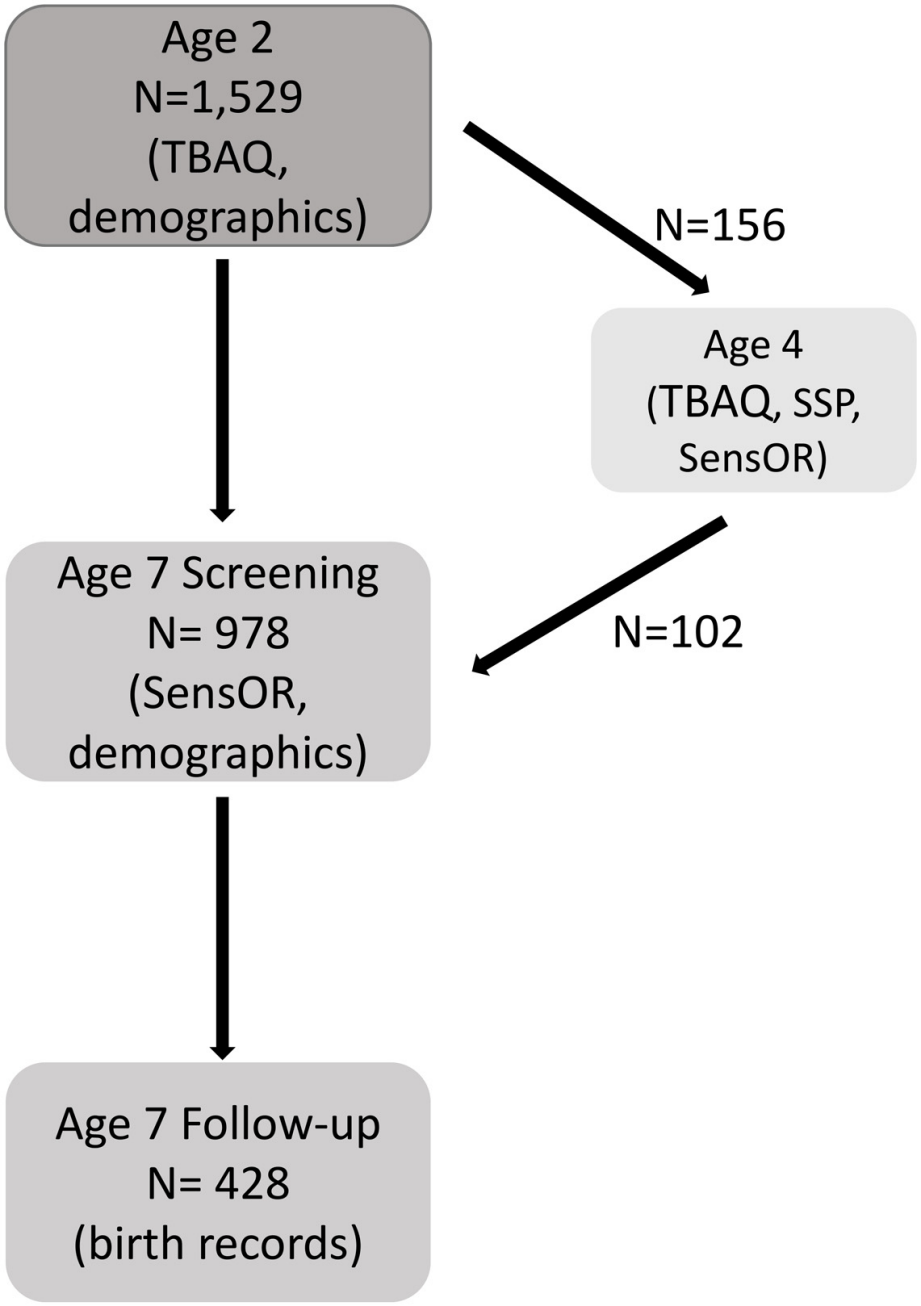
Sample size and inclusion criteria for each wave of data collection.

The sample comprised families of young twins recruited for the Wisconsin Twin Project, a statewide, birth register-based panel [48,49]. The names and contact information for all families were provided by the state for birth years 1998 through 2004. Analyses are based on three waves of data collection (see Fig 1). First, 2,125 families were invited to participate in a study of toddler temperament when twins were approximately two years old. Two-thirds of families responded, 1,881 (88%) of whom agreed to participate. Parents completed phone interviews and/or mailed questionnaires. Due to incomplete or unreturned questionnaires, sensory over-responsivity assessments were a completed by 1,529 (81%) primary caregivers for a total of 3,058 children. Sensory experiences were measured using the revised Toddler Behavior Assessment Questionnaire.

Second, at age seven years, twins were screened for behavioral problems via telephone interview with the primary caregiver with the goal of “enriching” a sub-sample with behavior problem symptoms for an in-depth follow-up. Twin pairs were selected for follow-up if at least one member scored >1.5 SD above the mean on at least one of the following scales: depression, anxiety, overanxious, aggression, oppositional defiance, conduct disorder, inattention, or impulsivity scales. A group of low symptom comparison participants (scoring below the mean for all scales) was also selected for follow-up. All cotwins of children selected for follow-up were included, regardless of cotwin risk status (at-risk, control, or neither). During the screening interview, sensory over-responsivity was assessed using the Sensory Over-responsivity Inventory but sensory symptoms were not used for selection purposes. A subset of 489 families (*N* = 978 children) who participated in the toddler studycompleted the behavioral screening at age seven years (50.2% female; *M* = 7.4 years, *SD* = .62 years, range 6–11 years), and 214 families (*N* = 428 children) were enrolled in the follow-up study. The remaining children were too old (N = 201 pairs) or too young (N = 861 pairs) for behavioral screening, were unreachable (N = 831), declined to participate (N = 61 pairs), or were enrolled in a different study (N = 126). The sample was 89.7% white, non-Hispanic, 3.9% Hispanic, 3.3% African American, and 3.1% other. Mother’s had on average 15.1 years of education (SD = 2.3). Father’s had on average 14.8 years of education (SD = 2.5). Income level was divided into categories ranging from $10,000 or less to over $200,000. Median family income was $50,000-$60,000.

Finally, to measure convergent validity of assessments across our target ages (ages two and seven years), we analyzed data from a small pilot study (N = 156 children; 62% female) that included multiple assessments of sensory experiences, including the assessments used at age 2 and age 7 years. At age 4 years, Children who had previously screened positive (i.e., top 25% on auditory or tactile subscales) for sensory over-responsivity in the toddler study, control children matched on age and gender to the target child, and their unselected cotwins were enrolled in a pilot study of sensory over-responsivity. Two-thirds of the families who participated in the pilot study went on to participate in behavioral screening at age seven years (N = 102 children). The remaining children were too old (N = 44) or declined to participate (N = 10) at age seven years.

### Ethics statement

At each assessment, parents of the children provided written informed consent following appropriate ethical guidelines, and the protocol was approved by the Social and Behavioral Sciences IRB at the University of Wisconsin–Madison.

### Measures and procedures

#### Age 2 assessment

Mothers and fathers completed a revised version of the Toddler Behavioral Assessment Questionnaire [50] when their offspring were two years of age (*M* = 26.4 months, *SD* = 3.0 months). The TBAQ is a caregiver-report temperament measure that assesses temperamental dimensions for 18- to 36-month olds. We used primary caregiver (98% mothers) report on a 120-item version with the following subscales: Activity Level, Anger, Attention, Inhibitory Control, Interest, Object Fear, Smiling/Laughter, Sadness, Social Fear, Soothability, and Sensory Over-responsivity. Parents reported how often they observed the specified behavior during the past month on a 1 (*never*) to 7 (*always*) Likert scale. For each subscale, scores were averaged across items.

The Sensory Over-responsivity subscale consists of ten items evenly split between auditory and tactile over-responsivity. The item content of the sensory over-responsivity scale of the TBAQ (see Table 1) is similar to corresponding parts of the widely used Short Sensory Profile [28]. The auditory and tactile symptom subscales showed moderate—for 5-item scales—internal consistencies (*α* = .67 and .57, respectively). The combined scale had a reliability of *α* = .70. These scales have been used previously to examine the genetic and environmental underpinnings of SOR symptoms [23,51]. While no specific cut-off criteria have been established to distinguish children at-risk for SOR from typically developing children, we relied on previous work [23,29] to designate any child in the top 5% on *either* the tactile *or* auditory subscales of the TBAQ as being at-risk for a diagnosis of SOR. This cut-point corresponds to a mean score of 4.6 on the tactile and 4.2 on the auditory subscale.

**Table 1:** Sensory over-responsivity items from the Toddler Behavioral Assessment Questionnaire (TBAQ).

Tactile	Auditory
When touching a new object, how often did your child seem child seem concerned by how smooth or rough the texture was?	How often did your child react noticeably when a low-pitched sound started suddenly (such as an air conditioner, a heating system, a refrigerator, or a vacuum from another room)?
How often did your child object to changes in articles of clothing that fit snuggly or tightly, (for example, putting on a hat, wearing gloves, getting new shoes)?	How often did your child seem to be alarmed when s/he heard sirens (such as a police, fire, or ambulance siren) in the distance?
How often did your child object to scratchy clothing fabrics such as wool?	How often did your child ask or gesture for the volume of loud music, radio, or TV to be lowered?
How often did your child refuse to touch a sticky or gooey substance (for example, shaving cream, mayonnaise, toothpaste, mud)?	How often did your child seem overly sensitive to, or irritated by, certain sounds, voices or music?
How often did your child object to the feeling of a comb moving through her/his hair or toothbrush touching her/his gums?	How often was your child distracted by background sounds that do not bother most other people?

Parents indicated frequency of observing each item in the past month on a scale from never (1) to always (7).

#### Convergent validity measures at Age 4

Mothers completed the TBAQ, Short Sensory Profile [28], and the Sensory Over-Responsivity Inventory (see below), as well as other questionnaires detailing family demographics, child health, pregnancy complications, maternal depression, and maternal sensory symptoms.

#### Age 7 assessment

Primary caregivers (98% mothers) completed the Sensory Over-Responsivity Inventory (SensOR) [27] separately for each twin at age seven years (*M* = 85 months, *SD* = 5.9 months). Caregivers indicated (yes or no) if their child was bothered by a particular sensation (e.g. mud on hands, brushing teeth; sound of appliances running). Thirty-one items assessed tactile sensitivity and 23 items assessed auditory sensitivity. We summed items to create tactile and auditory sensitivity symptom counts. Both subscales demonstrated good internal consistency (*α* = .84 and .83, for tactile and auditory subscales respectively). No empirically validated cut-off score, or threshold for predicting a true case, has been established for the SensOR. In consultation with the measure's author, we considered children at-risk for SOR if primary caregivers endorsed 8 or more symptoms of tactile defensiveness or 4 or more symptoms of auditory defensiveness; groups scoring at or above these thresholds represented the top 10% of participating children in each domain.

#### Birth and demographic information

Gestational age and birthweight were obtained from hospital birth records and/or maternal report at age seven. Correlations between birth record and maternal report were high (*r* = 0.89, *p* < .001 for gestational age and *r* = 0.97, *p* < .001 for birth weight). The primary caregiver reported parent education (mother and father separately), family income, and twins’ race/ethnicity at both ages. Socioeconomic status (SES) was calculated by averaging across standardized parent years of education and income. SES measures at ages two and seven years were highly correlated (*r* = .91).

### Data Analysis

Because two different measures were used to assess symptoms of SOR at the target ages, we used the age four subsample to examine convergent validity between these assessments. We then examined the consistency in screening positive for being at-risk for a diagnosis of SOR across ages two and seven years. Children were assigned to one of four symptom trajectories based on risk status at two and seven years. We used general linear models to test for mean differences among the trajectory groups on a variety of measures. Finally, we used logistic regression and general linear models to determine if birth and temperament risk factors predicted symptom trajectories or moderated the stability of SOR symptoms over time. A thorough investigation of the genetic underpinnings of SOR trajectories is beyond the scope of this paper. Therefore, all analyses controlled for clustering within families. Separate genetic analyses of SOR symptoms at ages two and seven years have been published elsewhere [22,23].

### Missing Data

Children who lacked age seven sensory data were excluded largely due to child age, as noted above. Children who participated at age seven years did not differ on SES (*t* = 1.3, *p* = .18), age at time of assessment (*t* = 1.3, *p* = .17) or gender (Fisher’s exact test = .21) compared with children who did not participate at age seven years. Children who participated at age seven years had higher tactile and auditory over-responsivity scores at age two years than those who did not participate at age seven, but the effect sizes were modest (Cohen’s *d* = .15 and .08 respectively). Less than one percent of participants were missing one or more items on the SenSOR. Forty-four percent of participants were missing one or more items on the TBAQ. Two items, “how often child alarmed when heard siren” and “how often child objected to scratchy clothes,” which were often rated as “not applicable,” accounted for 98% of the missing items. Children with complete data did not differ from children missing one or more TBAQ items on gender (Fisher’s exact test = .05), SES, age at either assessment, or sensory over-responsivity scores at either assessment (*t*s ranged from 0.9–1.4, *p*s>.10)

Missing values are common in social science research. The more items or questionnaires involved in an analysis, the more likely that any given subject is missing one or more items. Multiple imputation provides a flexible mechanism for dealing with item-level missingness when data are missing at random because it separates the missing data handling from the analysis [52]. Multiple imputation uses all variables with missing data as well as other related variables (e.g., gender and age). Each variable is regressed on all other variables to produce a predicted value for the missing data point (plus a random error term). The process is repeated until the desired number of “complete” datasets are generated. The analysis is then repeated for each imputed dataset and the results are combined across datasets [53]. Where scale scores are used in the place of individual items, Gottschall et al. [52] recommended imputing at the item level prior to creating scale scores for each imputed dataset.

For each analysis, we used the SAS procedure MI to generate 15 imputed datasets using only the item-level variables [53]. We used MIANALYZE to aggregate statistics across datasets (SAS Institute, 2011). All analyses were based on multiple imputation unless otherwise indicated and conducted using SAS software. A comma delimited file of the original (non-imputed) de-identified data used in these analyses and a list of variable labels and formats are available in supporting information files S1 Appendix and S2 Appendix, respectively.

## Results

### Screening positive for sensory over-responsivity

**Fig 2:**
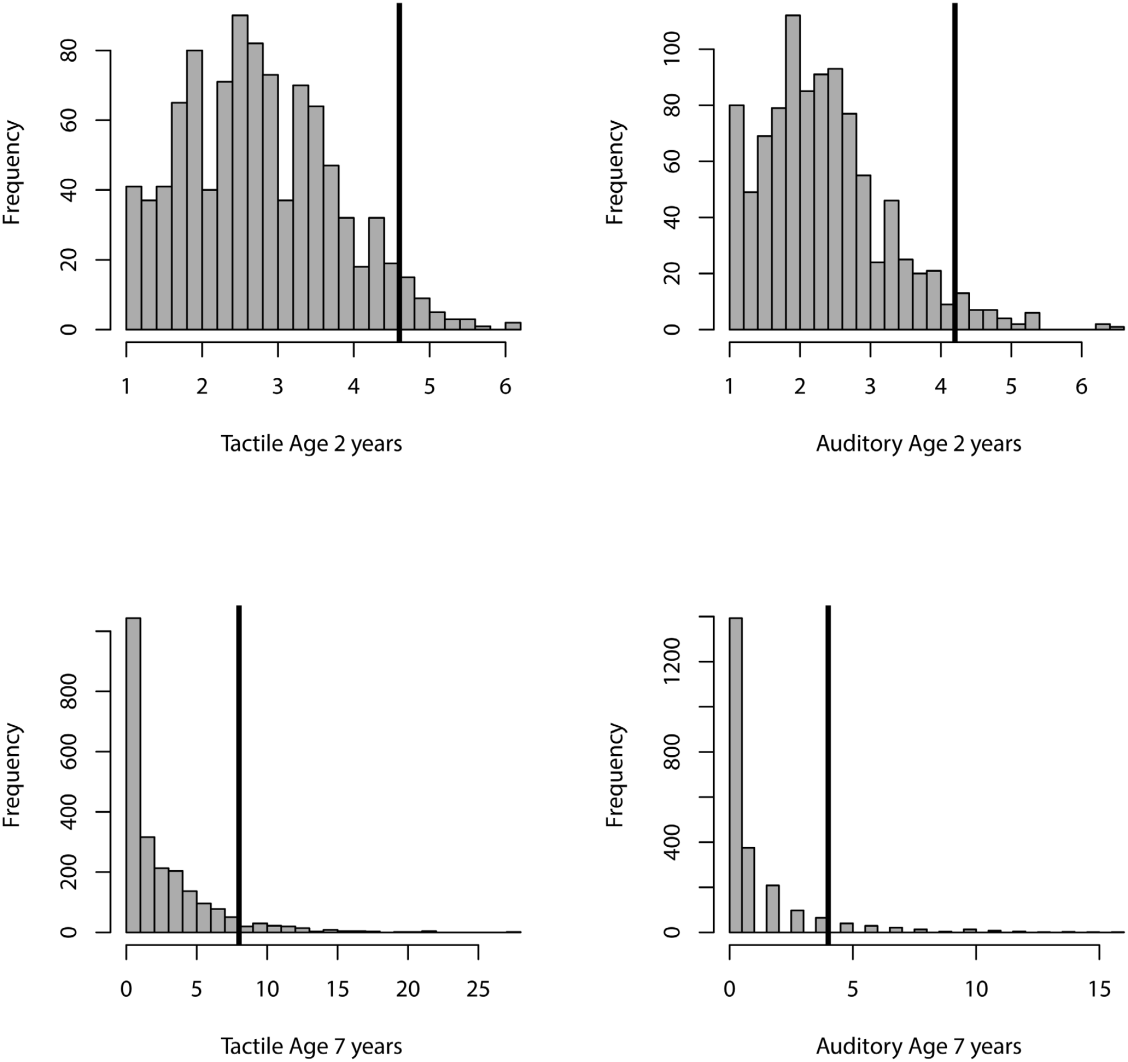
Histogram of tactile and auditory sensor over-responsivity at age two years (top row) and age seven years (bottom row). Vertical black bars indicate the cut-point for each SOR measure.

Using the cut-off scores described above, 294/3058 (9.4%) of children screened positive for SOR at age two years, and 142/978 (14.5%) children screened positive at age seven years. Histograms for each of the SOR measures and their cut-points are shown in Fig 2. Roughly equal numbers of males and females screened positive for SOR in toddlerhood (152 girls, 142 boys, OR = .93, 95%CI 0.7–1.2, p = .56). In contrast, more boys than girls screened positive for SOR at age seven years although the difference was only marginally significant (60 girls, and 82 boys; OR = 1.2, 95%CI 1.0–1.6, p = .10). Age at time of assessment and SES did not predict risk status at either assessment.

Primary caregivers participating in the age four sensory processing study completed both the TBAQ and SensOR questionnaires concurrently. We used this subsample to examine the equivalency of the two measures and the cut-off scores used to identify at-risk individuals. There were no missing items at this age so multiple imputation was unnecessary. The concurrent correlation across measures were .58 for tactile and .38 for auditory over-responsivity. Both TBAQ and SensOR measures were correlated with the Short Sensory Profile (*r* = .45 and .46 for auditory over-responsivity, *r* = .59 and .68 for tactile over-responsivity, respectively). Roughly equal numbers of children were identified as at-risk based on the TBAQ (27%) and SensOR (31%); 50% of children identified as at-risk on either measure were identified as at-risk on both measures. After standardizing all measures, SensOR symptom counts significantly predicted TBAQ ratings (b = .65, *t* = 8.0, *p* < .001 for tactile and b = .42, *t* = 4.1 *p* < .001 for auditory). We used the age four regression equation to obtain predicted TBAQ scores at age seven and then applied the TBAQ cut-points from age two years. Nine percent of children were identified as at-risk for SOR based on these pseudo-TBAQ scores. All of these children were identified as at-risk based on the SensOR cut-points as well. Therefore, we believe that the TBAQ and SenSOR provide adequate measures of the same underlying constructs.

### Characteristics of SOR trajectories

We divided children into a typically developing trajectory and three potentially at-risk trajectories. Seven hundred sixty-seven children (78%) never screened positive for SOR (Low symptom); 24 (2.5%) children screened positive in both toddlerhood and middle childhood (Chronic), double the number expected by chance given the “prevalence” rate at both ages (.094*.148*100 = 1.3%); 75 (8%) children only screened positive for SOR in toddlerhood (Remitted) and 112 (11.5%) children only screened positive for SOR at age seven (Late-onset). We turned again to the age four subsample to explore how children who screened positive across ages might differ from other groups on multiple measures of SOR. Mean scores on the TBAQ, SensOR, and Short Sensory profile (age four subsample only) for the low-symptom and three at-risk groups are presented in Table 2. We used eneral linear models to examine mean differences in these multiple measures of tactile and auditory responsivity symptoms across the four groups. Mean differences were all in the expected direction with the chronic risk group showing the most extreme scores, the low symptom group showing the least extreme scores, and the two intermediate risk groups in the middle. However, the differences generally only reached significance when comparing the chronic and low symptom groups. Except for the Short Sensory Profile auditory over-responsivity scale, children who screened positive for SOR at both ages two and seven years were perceived as having both higher tactile and auditory symptoms at four years than typically developing children. The few differences between the Remitted and Late-onset groups and the typically developing group were inconsistent across measures. However, the risk groups were small in the age four sample and substantial effect sizes were required for statistical significance. In addition to auditory and tactile sensitivity, the Short Sensory profile includes movement sensitivity. Children in the Chronic group scored lower (i.e. greater movement sensitivity) than children in the other three groups (see Table 2).

**Table 2:** Mean symptoms on three measures of SOR at age four years (total *N* = 102) by trajectory group.

	Low	Remitted	Late-Onset	Chronic
**Age 4 measures (range)**	*N* = 59	*N* = 18	*N* = 16	*N* = 9
**TBAQ tactile (1–7)**	2.4 (1.0)a	4.0 (1.3)a,b	3.1 (1.4)a	5.1 (0.9)b
**TBAQ auditory (1–7)**	2.3 (1.1)a	3.3 (0.9)b	2.8 (1.1)a,b	3.8 (0.7)b
**senSOR tactile (0–30)**	2.9 (2.6)a	4.4 (3.1)a	5.8 (3.2)a,b	8.9 (5.1)b
**senSOR auditory (0–22)**	0.9 (1.4)a	1.9 (2.1)a,b	1.8 (1.7)a,b	3.0 (2.5)b
**SSP tactile (1–5)**	4.4 (0.4)a	4.1 (0.6)a,b	3.8 (0.7)b	3.2 (0.8)b
**SSP auditory (1–5)**	4.1 (0.6)a	3.2 (0.7)b	3.4 (0.7)b	3.5 (0.7)a,b
**SSP movement (1–5)**	4.5 (0.6)a	4.4 (0.8)a	4.2 (1.1)a	3.1 (1.3)b

Means that do not share superscripts (a or b) are different at p < .05; TBAQ = Toddler Behavior Assessment Questionnaire; senSOR = Sensory-Over Responsivity Inventory; SSP = Short Sensory Profile; *low* scores on the Short Sensory Profile are indicative of greater sensitivity.

**Fig 3:**
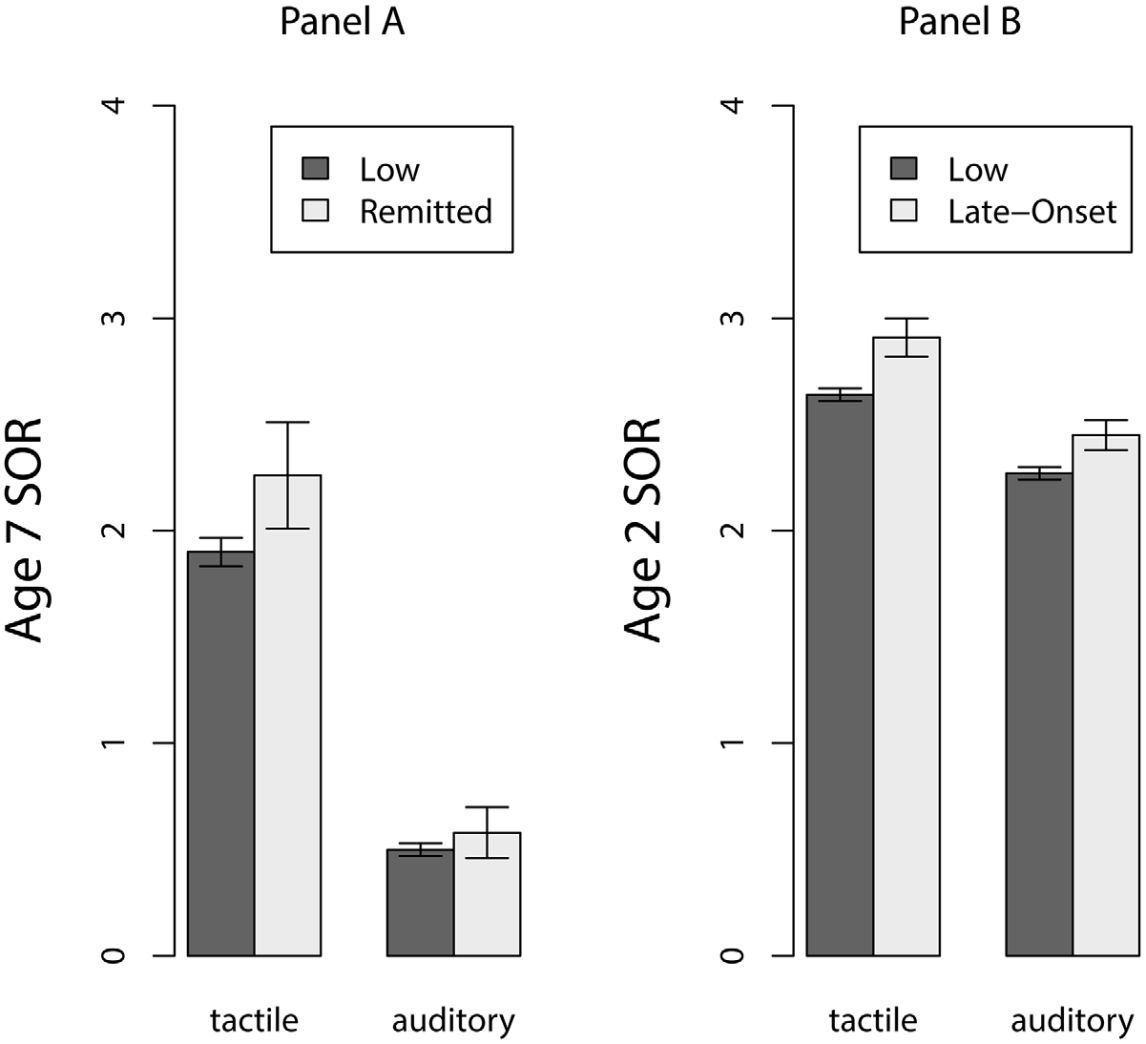
Mean sensory over-responsivity. Panel A: Age seven Remitted vs. Low symptom. Panel B:\. Age two Late-onset vs. Low symptom groups.

Children in the Remitted group may be elevated in their sensory symptoms at age seven even if they failed to score at or above threshold at that age. Similarly, children in the Late-onset group may have elevated scores at age two that fall just short of the at-risk cut-off score. For another perspective on continuity that is not so dependent on the cutoff scores, we tested for mean differences in SOR symptoms at age seven years between children in the Remitted and Low symptoms groups (Fig 3, Panel A). Similarly, we tested for mean differences in TBAQ symptoms at age two years between children in the Late-onset and Low symptom groups (Fig 3, Panel B). Children in the Remitted group scored approximately .10 standard deviations higher on auditory and tactile SOR at age seven years than the Low-symptom group, but the difference did not reach significance (*t* = 1.5, *p* = .15 tactile SOR and *t* = 0.7, *p* = .52 auditory SOR). In contrast, children in the Late-onset group scored approximately .25 standard deviations higher on tactile and auditory SOR symptoms at age two than the Low symptom group (*t* = 7.0, *p* < .0001 tactile SOR and *t* = 2.4, *p* = .01 auditory SOR).

### Predictors of SOR trajectories

We used logistic regression to predict SOR trajectories from age of assessment, SES, gender, and birth characteristics, either gestational age or low birth-weight status (<5.5 lbs) and temperament dimensions using the Low-symptom group as the comparison group. Due to collinearity between gestational age and birth weight we could not examine the contributions of one controlling for the other. SES, age at assessment and gender did not predict trajectory group membership. An increase of one week in gestational age in this sample decreased the odds of being in the chronic SOR group (OR = 0.87, 95%CI = 0.78–0.98, *p* = .03), and being low birth-weight increased the odds of being in the chronic group at trend level (OR = 2.47, CI% = 0.98–5.29, *p* = .05). Neither gestational age nor low birthweight status predicted membership in the Remitted or Late-onset groups. Higher scores on temperament dimensions of fear, sadness, anger, and activity level increased the odds of being in the Remitted or Chronic groups while higher scores on the dimension soothability decreased the odds of being in the Remitted or Chronic groups (see Table 3). None of the temperament scales were significant predictors of late-onset SOR, nor did temperament distinguish between the Remitted and Chronic groups.

**Table 3:** Age two temperament predicting sensory over-responsivity trajectory group and correlations with sensory over-responsivity at age seven years.

	Odds Ratio (95% CI)			*r*	
Age 2 Temperament	Remitted	Late-onset	Chronic	Tactile	Auditory
**Object fear**	3.6** (2.7–4.9)	1.1 (0.8–1.4)	5.0*** (3.0–8.1)	.14**	.13***
**Social fear**	1.4** (1.1–1.7)	1.0 (0.8–1.2)	2.5** (1.6–3.9)	.08**	.07*
**Sadness**	2.3*** (1.7–3.2)	1.1 (0.8–1.4)	2.6** (1.5–4.5)	.12***	-.01
**Attention**	1.0 (0.7–1.3)	1.1 (0.9–1.4)	0.8 (0.4–1.3)	-.03	.04
**Soothability**	0.6*** (0.4–0.8)	0.9 (0.7–1.2)	0.4** (0.3–0.7)	-.14***	-.01
**Inhibitory Control**	0.8 (0.6–1.1)	0.8 (0.6–1.0)	0.7 (0.4–1.2)	-.13***	-.06*
**Anger**	1.9** (1.4–2.5)	1.1 (0.9–1.4)	1.9** (1.2–3.0)	.15*	.03
**Activity Level**	1.5* (1.1–2.1)	1.2 (0.9–1.5)	1.6 (1.0–2.7)	.08*	.04

Low-symptom trajectory (*N* = 767) is the comparison group. Sample sizes for trajectory groups are *N* = 767 Low, *N* = 75 Remitted, *N* = 112 Late-onset, *N* = 24 Chronic.

*p < .*05*,

**p < .*01*,

***p < .*001*.

### Continuous SOR symptoms

We observed no gender or zygosity differences in mean levels of symptoms of SOR at either age two or seven years. Despite the narrow age range at the time of assessment, age was positively correlated with auditory over-responsivity symptoms at age two years (*r* = .10, 95% CI: .04-.17, *p* = .002). Age did not correlate with tactile over-responsivity symptoms at age two years or tactile or auditory over-responsivity symptoms at age seven years. SES was correlated with age two auditory (*r* = -.10, 95% CI:-.20-.00, *p* = .05) and age two tactile (*r* = -.18, 95% CI:-.29—.06, *p* = .01) over-responsivity. SES was not correlated with age seven auditory or age seven tactile over-responsivity.

Longitudinal correlations were relatively modest, *r* = .22 (95% CI: .15 to .28, p < .001) for tactile symptoms and *r* = .11 (95% CI: .05 to .18, p < .001) for auditory symptoms. Given that we found much higher concurrent correlations in the age four subsample, these low correlations are likely due to changes in sensory sensitivity rather than changes in the instrument alone.

### Moderation of symptom stability

**Fig 4:**
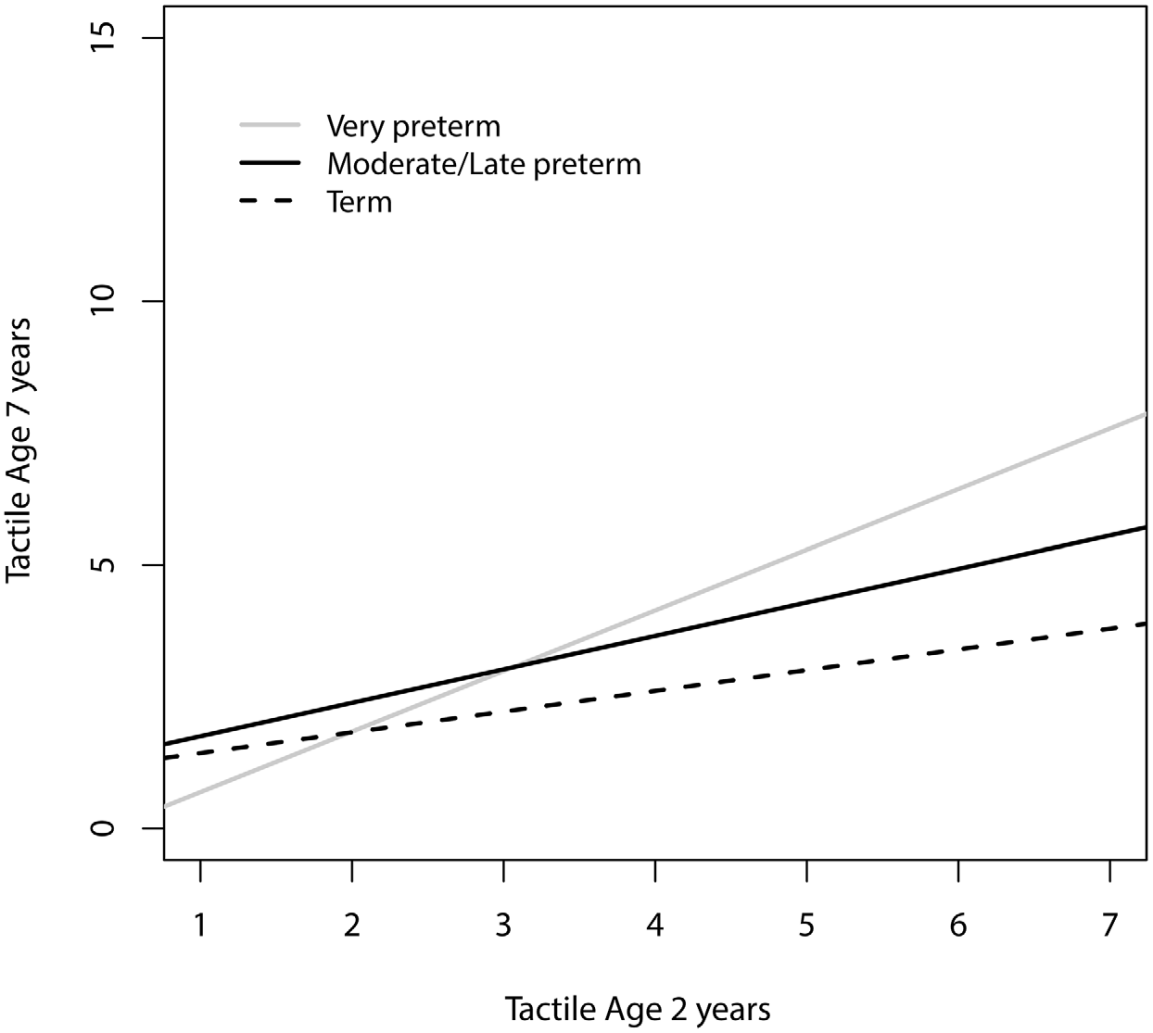
Tactile over-responsivity was more stable from age two to seven years for infants born very preterm.

**Fig 5:**
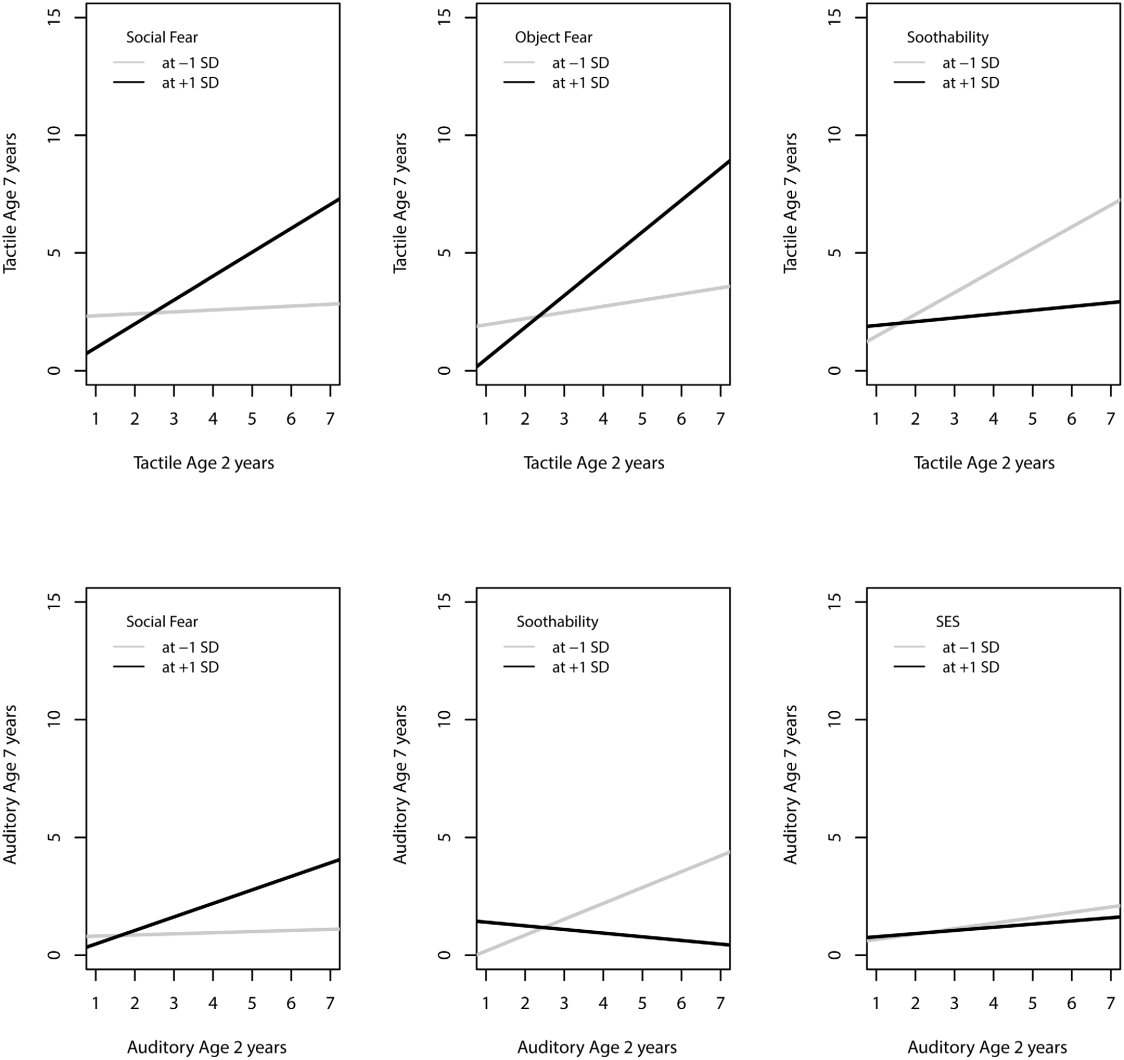
Regression of age 7 Sensory over-responsivity symptoms on age 2 symptoms. Black line represents children who scored 1 SD above the mean on emperamental fear or temperamental soothability or SES. Gray line represents children who scored 1 SD below the mean.

A series of general linear regressions determined if SES, gender, prematurity/ low-birth weight or temperament moderated the stability between early and later sensory over-responsivity across the spectrum of symptom severity. Gender did not moderate the relationship between SOR at age two and seven years. We found a significant interaction between gestational age and tactile over-responsivity (Fig 4) such that the earlier a child is born, the more strongly early tactile symptoms predict later tactile symptoms (*t* = 3.2, *p* = .002). We found a significant interaction between SES and auditory over-responsivity, such that early auditory symptoms were more predictive of later auditory symptoms among families with lower SES (*t* = -2.34, *p* = .02); however, the effect was quite small (Fig 5). Finally, we found significant interactions between temperament dimensions fear and soothability and sensory over-responsivity. Early childhood tactile over-responsivity was more highly predictive of later tactile over-responsivity among children who were more fearful (*t* = 4.1, *p* < .0001 for object fear and *t* = 2.8, *p* = .006 for social fear) and less soothable (*t = 2*.*1*, *p* = .04) in toddlerhood (Fig 5). Similarly, early auditory over-responsivity was more predictive of later auditory over-responsivity among children who were more fearful (*t* = 2.2, *p* = .03 for social fear) and less soothable (*t* = 2.1, *p* = .04).

## Discussion

We examined the developmental course of the most prevalent subtype of sensory modulation disorder, sensory over-responsivity, from toddlerhood to middle childhood and as well as potential contributors to the stability of sensory symptoms across development in two ways. First, since most children exhibited few symptoms of SOR. We created trajectory groups based on risk status at ages two and seven years to examine characteristics of the high-scoring children. Second, to gain insight into the stability of SOR across the spectrum of symptom severity, we also examined stability, and potential correlates thereof, in continuous measures of sensory symptoms.

In many children, SOR symptoms appear to be transient across development. Although the rate of children at-risk for SOR at each age was similar to rates reported in previous cross-sectional studies of similarly aged children [29,35], only a small subset of children experienced high levels of SOR symptoms at both ages. We think that the 2.5% estimate for the percentage of children who experience high levels of symptoms across development is a better benchmark for upper-limit prevalence of a SOR subtype of SMD than the higher estimates in the literature that are based on single age assessments.

Scoring in the at-risk range in toddlerhood in particular may net a mix of children with a propensity towards an underlying sensory problem and children experiencing a lag in otherwise typical development. Most of the children who scored in the at-risk range at age two years were indistinguishable from low-symptom peers by age seven years both on sensory symptoms and birth characteristics. This is not unexpected given that screening for low base rate phenotypes inevitably leads to false positives [54]. Characteristics of children identified as chronically at-risk included greater sensory sensitivity at both ages than low symptom peers or children whose symptoms fluctuated. Children in the chronically at-risk group experienced greater motion sensitivity (e.g. anxiousness or fear of tumbling/falling) on average at age four years than children with low symptoms or children whose symptoms fluctuated over time. Motor dysfunction is one of the key features of sensory modality disorder as identified by Ayres [4], and children with movement difficulties also tend to have elevated SOR symptoms (see [55] for a review). Chronically at-risk children were also more likely to have low birthweight or be born prematurely. Researchers should be cautious when identifying young children with SOR when assessment includes a single time-point without incorporating measures of other risk factors.

In contrast, given the mean group differences between the late-onset and low symptom groups at age two years, at least some children who were not identified as at-risk until middle childhood were already exhibiting a non-clinical elevation in sensory over-responsivity in toddlerhood. Yet, children in the late-onset group did not differ from low-symptoms peers or from children who were only at-risk in toddlerhood on temperament and birth characteristics. Possibly, children in the late-onset group develop sensory symptoms via a different mechanism than children in the chronic at-risk group.

Several temperament dimensions were associated with increased odds of being in the chronic or remitted groups but not the late-onset group. In other words, *early* temperament dimensions predicted being identified as at-risk in toddlerhood only. In our earlier work, we reported moderate correlations between temperament and auditory and tactile sensitivity in toddlerhood [23,51]. One possible explanation for these findings is that the relationship between temperament and SOR may diminish over time. A second explanation is that the relationship between temperament and SOR at age two may reflect shared measurement variance. Given that, at age two, the sensory scale was imbedded in the temperament questionnaire, shared variance likely contributes at least in part to the association between SOR and temperament. However, Keuler et al [56] also reported common genetic influences on temperament and SOR symptoms. That is, the cross-trait association between temperament and SOR symptoms was stronger for identical twins (who share 100% of their genes) than fraternal twins (who share on average 50% of their segregating genes). We would not expect shared measure variance to depend on twin type [46]. Thus, a third explanation is that sensory function is more intertwined with the affective processes involved in temperamental individuality during early development than later childhood, just as sensory function is key to cognitive function during early development [57].

Gestational age, birthweight and temperament contributed to the stability of tactile, and, to a lesser extent, auditory over-responsivity symptoms across the spectrum of symptom severity. Early tactile symptoms were more strongly associated with later tactile symptoms among children with a low gestational age or with a more fearful or less soothable temperament. Early sensory symptoms were more strongly associated with later sensory symptoms among children who were less soothable and more fearful. Thus, caregivers and researchers should pay special attention to children who present with sensory symptoms in the presence of prematurity or certain extreme temperaments as they are more likely to persist in their symptoms. It is unclear why temperament moderates tactile over-responsivity to a greater extent than auditory over-responsivity. Although many studies combine auditory and tactile sensitivities, correlations between them were only moderate. Very few children scored high on both tactile and auditory over-responsivity. In addition, we previously showed that individual variation in auditory and tactile sensitivity can be attributed to genetic factors unique to the two modalities, as well as genetic factors common to both [23].

### Study limitations

The first limitation of this study is that, without a gold standard of assessment or published norms, any particular cut-point on a scale of SOR is somewhat arbitrary. For this study, we chose cut-points in consultation with the measures’ authors. That prevalence rates in our study match those reported in previous studies bolsters our choice of cut-point. Second, SOR is not as familiar to parents as, say, anxiety or impulsivity. Also, parents may adjust the child’s environment in response to sensory dysfunction, thus reducing overt symptoms and, in a sense, then under-reporting symptoms. Other parents may under-report symptoms because they fail to discern the link between sensations and the child’s reactions. On the other hand, parents may over-report symptoms if they have limited experience with what the extremes of sensory reactions can be; that is, they may inflate the number of symptoms by reporting what are really moderate responses as extreme. These problems are not specific to SOR but reflect possible biases that are inherent in any parental report questionnaire. These biases may lead to misclassification of risk status. As awareness of sensory dysfunctions begins to permeate society, we expect parents to become more accurate reporters of their children’s sensory function. Third, while representative of families in Wisconsin, our participants were nevertheless mainly white, middle class families, which limits the generalizability of results. Finally, we emphasize that ours is a study of symptoms rather than of impairment; we do not know the extent to which SOR symptoms hampered adaptive functioning, or, the extent to which SOR symptoms might be associated with unrecognized strengths.

## Conclusions

Approximately 10–20% of typically developing children experience unpleasant reactions to ordinary tactile or auditory sensations, but for the majority of these children, such symptoms are transient. The small number of children (2.5%) who experience elevated symptoms across ages had more extreme symptoms and differed from their peers on other characteristics. This small, chronically affected group may represent the true sub-population of children at risk for a sensory processing disorder. Future work should investigate the daily functioning of this chronic group in more clinical detail.

## Supporting Information

S1 Appendix De-identified comma delimited data file with all original (non-imputed variables). (CSV)

S2 Appendix Text file of descriptive variable labels and formats. (TXT)
